# Performance of Stepwise Screening Methods in Identifying Individuals at High Risk of Type 2 Diabetes in an Iranian Population

**DOI:** 10.34172/ijhpm.2021.22

**Published:** 2021-05-05

**Authors:** Mojtaba Lotfaliany, Farzad Hadaegh, Mohammad Ali Mansournia, Fereidoun Azizi, Brian Oldenburg, Davood Khalili

**Affiliations:** ^1^Department of Biostatistics and Epidemiology, Research Institute for Endocrine Sciences, Shahid Beheshti University of Medical Sciences, Tehran, Iran.; ^2^Barwon Health, Geelong, VIC, Australia.; ^3^School of Population and Global Health, University of Melbourne, Melbourne, VIC, Australia.; ^4^Institute for Mental and Physical Health and Clinical Translation (IMPACT), Deakin University, Geelong, VIC, Australia.; ^5^Prevention of Metabolic Disorders Research Center, Research Institute for Endocrine Sciences, Shahid Beheshti University of Medical Sciences, Tehran, Iran.; ^6^Department of Epidemiology and Biostatistics, School of Public Health, Tehran University of Medical Sciences, Tehran, Iran.; ^7^Endocrine Research Center, Research Institute for Endocrine Sciences, Shahid Beheshti University of Medical Sciences, Tehran, Iran.; ^8^WHO Collaborating Centre on Implementation Research for Prevention & Control of NCDs, University of Melbourne, Melbourne, VIC, Australia.

**Keywords:** Primary Prevention, Type 2 Diabetes, Stepwise Screening, Prediction Models, External Validation

## Abstract

**Background:** Recent evidence recommended stepwise screening methods for identifying individuals at high risk of type 2 diabetes to be recruited in the lifestyle intervention programs for the prevention of the disease. This study aims to assess the performance of different stepwise screening methods that combine non-invasive measurements with lab-based measurements for identifying those with 5-years incident type 2 diabetes.

**Methods:** 3037 participants aged ≥30 years without diabetes at baseline in the Tehran Lipid and Glucose Study (TLGS) were followed. Thirty-two stepwise screening methods were developed by combining a non-invasive measurement (an anthropometric measurement (waist-to-height ratio, WtHR) or a score based on a non-invasive risk score [Australian Type 2 Diabetes Risk Assessment Tool, AUSDRISK]) with a lab-based measurement (different cut-offs of fasting plasma glucose [FPG] or predicted risk based on three lab-based prediction models [Saint Antonio, SA; Framingham Offspring Study, FOS; and the Atherosclerosis Risk in Communities, ARIC]). The validation, calibration, and usefulness of lab-based prediction models were assessed before developing the stepwise screening methods. Cut-offs were derived either based on previous studies or decision-curve analyses.

**Results:** 203 participants developed diabetes in 5 years. Lab-based risk prediction models had good discrimination power (area under the curves [AUCs]: 0.80-0.83), achieved acceptable calibration and net benefits after recalibration for population’s characteristics and were useful in a wide range of risk thresholds (5%-21%). Different stepwise methods had sensitivity ranged 20%-68%, specificity 70%-98%, and positive predictive value (PPV) 14%-46%; they identified 3%-33% of the screened population eligible for preventive interventions.

**Conclusion:** Stepwise methods have acceptable performance in identifying those at high risk of incident type 2 diabetes.

## Background

**Key Messages**

**Implications for policy makers**

- Stepwise screening methods using non-invasive measurements in combination with lab-based measurements had acceptable sensitivity (up to 68%) and specificity (at least 70%) for identifying high-risk individuals for type 2 diabetes.
- Using stepwise methods can eliminate the need for lab measurements in about half of the screened population.
- Using stepwise methods can limit the proportion of the population who need preventive interventions in the screened population to less than 35%.
- Findings from this study can play a key role in designing diabetes prevention programs.
- Findings from this study can be used to optimize screening methods in current national programs for the prevention of non-communicable diseases.

**Implications for the public**

Type 2 diabetes is the most common chronic disease worldwide, causing a lot of morbidity and mortality every year. There is strong evidence showing that structured lifestyle interventions can prevent type 2 diabetes among individuals at high risk of type 2 diabetes. One of the main barriers to the implementation of structured lifestyle interventions in low- and middle-income countries (LMICs) is the lack of validated and reliable screening methods for these programs to identify high-risk individuals. In this study, we developed stepwise screening methods by combining a non-invasive measurement with a lab-based measurement; and assessed their performance in predicting the 5-year incidence of type 2 diabetes. We showed that our stepwise screening methods had very good performances in identifying high-risk individuals and utilizing them can potentially prevent a considerable amount of morbidity and mortality.

There is strong evidence showing that structured lifestyle interventions can prevent type 2 diabetes among individuals at high risk of type 2 diabetes.^1-5^ These interventions showed to be most effective in short terms (<5 years) and among individuals at high risk of developing type 2 diabetes.^1-3,5^ These lifestyle intervention programs have been integrated to the healthcare system in a few high-income countries such as Finland,^6,7^ the United States,^8^ and Australia,^9^ as an effective component of the public health system for reducing the burden of type 2 diabetes. One of the main barriers for the implementation of structured lifestyle interventions in low- and middle-income countries (LMICs) is the lack of validated and reliable screening methods for these programs to identify high-risk individuals.^10,11^

 Lifestyle intervention programs used a wide range of screening methods for identifying high-risk individuals including non-invasive measurements (such as body mass index [BMI]), lab-based tests (such as fasting plasma glucose [FPG]), or a combination of them (stepwise screening methods).^1,2^ Several studies compared the performance of different screening methods for identifying high-risk individuals in the structured lifestyle interventions^9,12,12^; showing the superiority of the stepwise methods that combine a non-invasive prediction model with a simple blood test such as FPG^9,12^ or a lab-based prediction model.^13^ The stepwise screening methods for identifying high-risk individuals in the interventions for the prevention of type 2 diabetes were also recommended by the American Diabetes Association and National Institute for Health and Care Excellence guidelines.^14,15^ Despite this, there are few studies in LMICs that assessed the performance of different stepwise potential screening methods for identifying high-risk individuals that can be used in lifestyle intervention programs.

 Recently, we showed that non-invasive methods including several anthropometrics and non-invasive risk prediction models have acceptable discrimination for identifying those at high risk of type 2 diabetes in the Iranian population with waist-to-height ratio (WtHR) among anthropometrics and Australian Type 2 Diabetes Risk Assessment Tool (AUSDRISK) among non-invasive prediction models showing the best discrimination power (area under the curve (AUC) >0.70).^16-18^ Furthermore, we showed that two lab-based perdition models for type 2 diabetes namely Saint Antonio (SA)^19^ and Framingham Offspring Study (FOS) ^20^ have a high discrimination power (AUCs ≥0.78) in identifying those at high risk of type 2 diabetes in Iranian population ^21,22^. SA, FOS, and the Atherosclerosis Risk in Communities (ARIC) ^23^ lab-based prediction models were also showed to have high potentials to be used in routine clinical practice.^24^

 In this study, we aim to develop stepwise screening methods by combining a non-invasive measurement (WtHR or AUSDRISK) with a lab-based measurement (different cut-offs of FPG or predicted risk based on SA, ARIC, or FOS models); and to assess sensitivity, specificity, and positive predictive value (PPV) of those screening methods for predicting the 5-year incidence of type 2 diabetes. We also assessed validation, calibration, and usefulness of SA, ARIC, and FOS models for predicting the 5-year incidence of type 2 diabetes in different risk thresholds in Iranian population.

## Methods

###  Study Population

 Tehran Lipid and Glucose Study (TLGS) is a longitudinal study in a community-representative sample of Tehran, capital of Iran.^25^ Details of TLGS have been reported previously^25^; briefly, data collection was started in 1999-2001 on 15 005 individuals (phase 1) and all participants were re-examined triennially (phase 2 and later). Of 15 005 participants, 5630 individuals assigned to a community-wide lifestyle intervention program. In this study, only participants in the control arm and those aged ≥30 years were included (n = 4908). Moreover, 689 participants with type 2 diabetes at the baseline and 42 further participants with pregnancy were excluded. Out of 4177 eligible participants, 271 participants with no information on type 2 diabetes at baseline, 645 participants with no follow-up, 69 participants with no information on type 2 diabetes at follow-up waves, and 155 participants with total follow-up <5 years and without incidence of type 2 diabetes during the follow-up were excluded, leaving 3037 participants for the main analyses.

###  Clinical and Laboratory Measurements

 The details of clinical and laboratory measurements have been reported elsewhere.^25^ Participants were interviewed to obtain demographics, past medical history by completing a standardized and validated questionnaire. Physical activity level was assessed with the Lipid Research Clinic questionnaire. Anthropometric measurements were taken with shoes removed and the participants wearing light clothing. For measuring blood pressure, the participants remained seated for 15 minutes, then a qualified physician measured blood pressure two times after one more measurement for determining peak inflation level using a standard and calibrated mercury sphygmomanometer. A blood sample was drawn between 7:00 and 9:00 am into vacutainer tubes from all study participants after 12–14 hours overnight fasting. For the oral glucose tolerance test, 75 g anhydrous glucose was administered orally. FPG and two-hour postprandial plasma glucose (2hPG) were measured using an enzymatic colourimetric method with glucose oxidase. High-density lipoprotein cholesterol (HDL-C) was measured after precipitation of the apolipoprotein B containing lipoproteins with phosphotungstic acid. Triglycerides were assayed using glycerol phosphate oxidase.

###  Definition of Terms

 Type 2 diabetes was ascertained among participants who had FPG ≥7.0 mmol/L or 2hPG ≥11.1 mmol/L and/or were taking glucose-lowering medication. The event date was considered as the half-time between the first date that the type 2 diabetes was diagnosed and the last known disease-free date. BMI was calculated by dividing the weight in kilograms by the square of height in meters. Current smoking was ascertained in those participants who smoked cigarettes at least once a day or who smoked cigarettes occasionally. The family history of diabetes was defined as having at least one parent or sibling with diabetes.

###  Deriving Cut-Offs for Stepwise Screening Methods

 For non-invasive measurement in the stepwise methods, either WtHR with a cut-off of ≥ 0.55 or AUSDRISK with a cut-off of >15 has been used for this study. Both non-invasive measurements showed to have acceptable sensitivity (about 75%) but low specificities (about 50%) for identifying high-risk individuals in these cut-offs.^16-18^

 For FPG, cut-offs of 5.0 mmol/L (90 mg/dL), 5.3 mmol/L (95 mg/dL), 5.5 mmol/L (100 mg/dL), and 6.1 mmol/L (110 mg/dL) were used in stepwise screening methods. These cut-offs were selected based on the American Diabetes Association guideline, the World Health Organization (WHO) recommendations, or previous TLGS studies that showed a significant rise in the risk of development of type 2 diabetes from FPGs more than 5.0 mmol/L.^21,26,27^

 To derive cut-offs for risk predicted by lab-based risk prediction models, firstly, models were validated and calibrated for predicting the 5-year incidence of type 2 diabetes in Iranian population; decision curves were then used to identify the range of risk thresholds in which models can be used in stepwise screening methods. Four cut-offs for predicted risks within the range of those risk thresholds were derived to be used in stepwise screening methods.

 Thirty-two different stepwise screening methods were developed by coupling every possible pair of a non-invasive measurement (either WtHR or AUSDRISK) and a lab-based measurement (either FPG or a predicted risk based on lab-based risk prediction model).

###  Brief Description of Risk Prediction Models 

 A brief description of risk prediction models was provided in Supplementary file 1.

###  Statistical Analysis

####  Baseline Characteristics Summarization

 The baseline characteristics were summarized as mean (standard deviation, SD) values for continuous and frequencies (%) for categorical variables in those with and without type 2 diabetes after 5 years of follow-up. For continuous variables, we test if their distributions follow a normal distribution visually (ie, histograms, P-P plots, Q-Q plots) and statistically (comparing their skewness and excess kurtosis — kurtosis minus three). Since the blood level of triglycerides had a high skewness and excess kurtosis (skewness = 2.9 and excess kurtosis = 17.7), it was summarized by the median (interquartile range, IQR). Comparison of baseline characteristics between participants with different glycemic status was done by Student’s *t* test for continuous variables, the chi-square test for categorical variables, and Mann-Whitney U test for skewed variables. To correct for multiple comparisons in baseline characteristics comparisons, we used Bonferroni correction, setting the *P* value threshold for statistically significant equal to.002.

####  Missing Data Imputation

 Before validation and calibration of lab-based risk prediction models, single imputation was performed to impute missing values for variables with missing data. The imputation of missing values before the validation of risk prediction models has been recommended before.^28^ For imputing the missing values of hip circumference (n = 54), BMI (n = 54), waist circumference (n = 55), systolic blood pressure (n = 64), and HDL-C (n = 3), linear regressions and for imputing the missing values of physical activity (n = 51), family history of type 2 diabetes (n = 122) and smoking status (n = 49), logistic regressions, were fitted using age, sex, education status, self-report of hypertension, hyperglycemia, drug history for hypertension, diastolic blood pressure, 2h-PG, total cholesterol, and triglycerides, and dyslipidemia as axillary variables.

####  Models Calibration 

 To calibrate each prediction model, logistic regression was fitted with type 2 diabetes as the outcome and the linear predictor part as the offset variable to calibrate the risk prediction model intercept, developing a “calibrated-in-the-large” model^28^; furthermore, a separate logistic regression was fitted with type 2 diabetes as the outcome and the linear predictor part as the only predictor variable to derive the calibration slope and new intercept, developing a “recalibrated” model.^28^

####  Models Validation 

 To assess the discrimination power of the models, a receiver operating characteristic (ROC) curve was plotted and the AUC has been estimated for each risk prediction model and FPG. Calibration of the original, calibrated-in-the-large, and recalibrated models were assessed visually as well as using Hosmer-Lemeshow chi-squared; a Hosmer-Lemeshow Chi-square higher than 20 was defined as the clear evidence for lack of calibration.^29^ In line with guidelines from Transparent Reporting of a multivariable prediction model for Individual Prognosis or Diagnosis Initiative,^30^ we plotted predicted outcome probabilities (x-axis) against observed outcomes (y-axis) using a LOWESS (locally weighted scatterplot smoothing) line.

 To assess the range of risk thresholds in which the risk prediction models are useful, the net benefit of the models were plotted in the wide range of risk thresholds to draw decision curves.^31,32^ The net benefits of the risk prediction models were compared with the net benefits of two different scenarios without screening: treat-none and treat-all.^31,32^ The risk prediction models were useful in a risk threshold if the differences between the net benefit of the risk prediction model and no-screening methods were equal or higher than 0.01^31,32^; cut-off of 0.01 for the difference in the net benefit for minimal invasive screening methods has been suggested previously.^31,32^ Based on the range of useful risk thresholds in the decision curves, four cut-offs for predicted risk of type 2 diabetes were derived to be used in stepwise screening methods. As the supplementary analyses, all validation analyses were repeated in the two different subgroups of participants: those with WtHR ≥0.55 (n = 1466) and those with AUSDRISK >15 (n = 1557).

####  Stepwise Screening Methods Development/Assessment

 Thirty-two stepwise screening methods were developed by combining a non-invasive measurement (WtHR ≥0.55 or AUSDRISK >15) with a lab-based measurement (cut-offs of FPG and predicted risk based on each risk prediction model). Sensitivity, specificity, and PPV of stepwise methods for identifying those with 5-year incident type 2 diabetes were assessed. We also assessed the proportion of the screened population who need lab tests and the proportion of the screened population eligible for preventive interventions based on the stepwise screening methods. The proportion of the screened population who need lab tests is defined as the proportion of participants who identified as high risk in the first step of the screening method (WtHR ≥0.55 or AUSDRISK score >15, whichever appropriate based on the stepwise screening method). Based on our suggested screening methods, those with WtHR <0.55 or AUSDRISK score ≤15 does not need further investigations (ie, lab tests). The proportion of the screened population eligible for preventive interventions is defined as the proportion of participants who identified as high risk in both the first step (WtHR or AUSDRISK) and the second step (FPG or lab-based risk scores) of the screening method. Based on our suggested screening, lifestyle interventions are not recommended for those who do not found high-risk in both the first and the second steps of the screening method. Corresponding 95% confidence intervals (CIs) were derived using bootstrap methods (percentile confidence intervals).

####  Sensitivity Analyses

 In the sensitivity analyses, we further imputed the missing values of the type 2 diabetes status in those eligible participants who were excluded from the analyses due to missing data for type 2 diabetes at baseline examination or after 5 years of follow up using similar methods for imputation (n = 1140). We also repeated all analyses in participants with complete data (n = 2872). All analyses were performed using Stata statistical software (version 14 SE).

## Results

###  Baseline Characteristics 

 Of 3037 participants, 203 (6.7%) ones developed type 2 diabetes during 5 years of follow-up. Table 1 compares the baseline characteristics of participants by their type 2 diabetes status. Participants aged 46 years on average and 55% were women. The average WtHR and AUSDRISK score were 0.55 and 16, respectively, with 49% of participants having WtHR ≥ 0.55 and 51% of participants having AUSDRISK score >15. Those who developed type 2 diabetes within 5 years of follow-up were older, had lower education and higher levels of weight, BMI, WtHR, AUSDRISK score, systolic and diastolic blood pressure, FPG, 2hPG, pre-diabetes, total cholesterol, and triglycerides.

**Table 1:** Baseline Characteristics of Participants

**Characteristics**	**Total (n = 3037)**	**Diabetes Free (n = 2834)**	**Type 2 Diabetes (n = 203)**	***P*** **Value** ^a^
Age	45.86 (11.19)	45.57 (11.14)	49.93 (11.15)	<.001
Gender (%)				
Female	1685 (55.5%)	1564 (55.2%)	121 (59.6%)	.223
Education (%)				
Less than 6 years	1134 (37.4%)	1022 (36.1%)	112 (55.4%)	<.001
6-12 years	1507 (49.7%)	1432 (50.6%)	75 (37.1%)	
More than 12 years	390 (12.9%)	375 (13.3%)	15 (7.4%)	
Physically inactive (%)	2217 (74.2%)	2074 (74.4%)	143 (72.2%)	.498
Current smoking (%)	456 (15.3%)	429 (15.4%)	27 (13.6%)	.513
Weight (kg)	71.77 (12.18)	71.51 (12.09)	75.53 (12.85)	<.001
BMI (kg/m^2^)	27.33 (4.40)	27.19 (4.34)	29.39 (4.74)	<.001
Waist circumference (cm)	89.48 (11.02)	89.05 (10.93)	95.63 (10.43)	<.001
WtHR	0.55 (0.07)	0.55 (0.07)	0.60 (0.07)	<.001
WtHR ≥0.55	1466 (49.2%)	1318 (47.3%)	148 (75.5%)	<.001
AUSDRISK score	15.98 (6.00)	15.69 (5.90)	20.22 (5.88)	<.001
AUSDRISK score >15	1557 (51.3%)	1408 (49.7%)	149 (73.4%)	<.001
Systolic blood pressure (mm Hg)	119.57 (17.86)	118.88 (17.43)	129.37 (20.80)	<.001
Diastolic blood pressure (mm Hg)	78.68 (10.25)	78.42 (10.14)	82.38 (11.15)	<.001
FPG (mmol/L)	5.06 (0.54)	5.01 (0.51)	5.68 (0.61)	<.001
Postprandial plasma glucose (mmol/L)	6.10 (1.67)	5.98 (1.58)	7.85 (1.91)	<.001
Cholesterol (mmol/L)	5.54 (1.16)	5.52 (1.14)	5.82 (1.29)	<.001
HDL (mmol/L)	1.08 (0.28)	1.08 (0.28)	1.05 (0.26)	.081
Triglycerides (mmol/L)	1.70 (1.19, 2.42)	1.69 (1.16, 2.37)	2.12 (1.45, 3.04)	<.001

Abbreviations: BMI, Body mass index; WtHR, Waist-to-height ratio; AUSDRISK, Australian Type 2 Diabetes Risk Assessment Tool; FPG; Fasting plasma glucose; HDL, high density lipoprotein. ^a^ *P* value was calculated using Student’s *t* test for continuous variables and the chi-square test for categorical variables and Mann-Whitney U test for skewed variables. To correct for multiple comparisons in baseline characteristics comparisons, we used Bonferroni correction, setting the *P* value threshold for statistically significant equal to.002.

 Some observations have missing information for hip circumference (n = 54), weight (n = 54), BMI (n = 54), waist circumference (n = 55), WtHR (n = 55), AUSDRISK (n = 145), systolic and diastolic blood pressure (n = 64), and HDL-C (n = 3), physical activity (n = 51), family history of type 2 diabetes (n = 122), education status (n = 6), and smoking status (n = 49). The missing values were imputed for further analyses.

###  Validation of Lab-Based Risk Prediction Models

**Figure 1:**
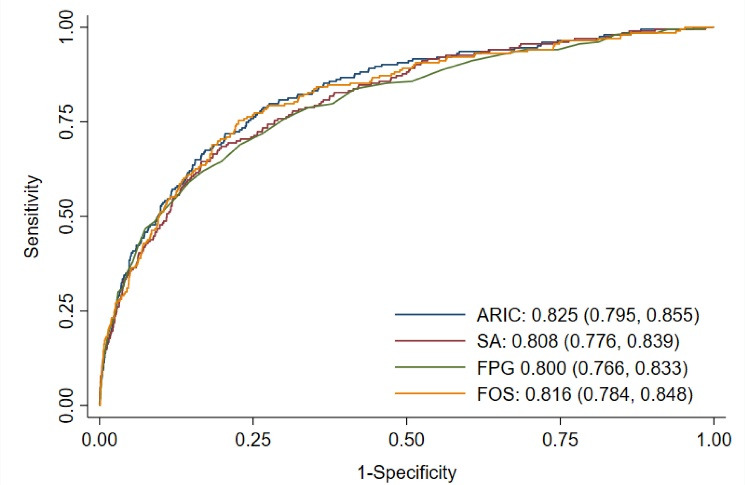
ROC curves. Abbreviations: ROC, Receiver operating characteristic; SA, Saint Antonio; FOS, Framingham Offspring Study; FPG, Fasting plasma glucose; ARIC, Atherosclerosis Risk in Communities.

 ARIC risk prediction model had the highest discrimination power (AUC = 0.83) as compared to SA (AUC: 0.81; *P* value = .004) and FPG (AUC: 0.80; *P* value = .015) but there was no significant difference between AUC of ARIC and FOS risk prediction models (AUC: 0.82; *P* value = .204) (Figure 1). Figure S1 shows the ROCs of the models in two different subgroups of participants who had WtHR ≥0.55 and AUSDRISK >15; in both subgroups, there was no statistically significant difference in discrimination power between risk prediction models and FPG.

 Table S1 compares the equations of original and calibrated risk prediction models. The calibration slopes ranged from 0.96 in SA model and 0.95 in ARIC model to 0.69 in FOS model. Figure S2 shows the calibration plot of the original, calibrated-in-the-large, and recalibrated models. While the original models for ARIC, SA, and FOS risk prediction models overestimated the risk (Hosmer-Lemeshow chi-squared ranged from 100 to ARIC to 1328 in FOS), the predicted and observed risks in recalibrated models were comparable showing a reasonably good calibration for all three risk prediction models (Hosmer-Lemeshow chi-squared <20). Similar findings were observed in those with WtHR ≥0.55 and AUSDRISK >15 (Figure S3, Supplementary file 2).

**Figure 2:**
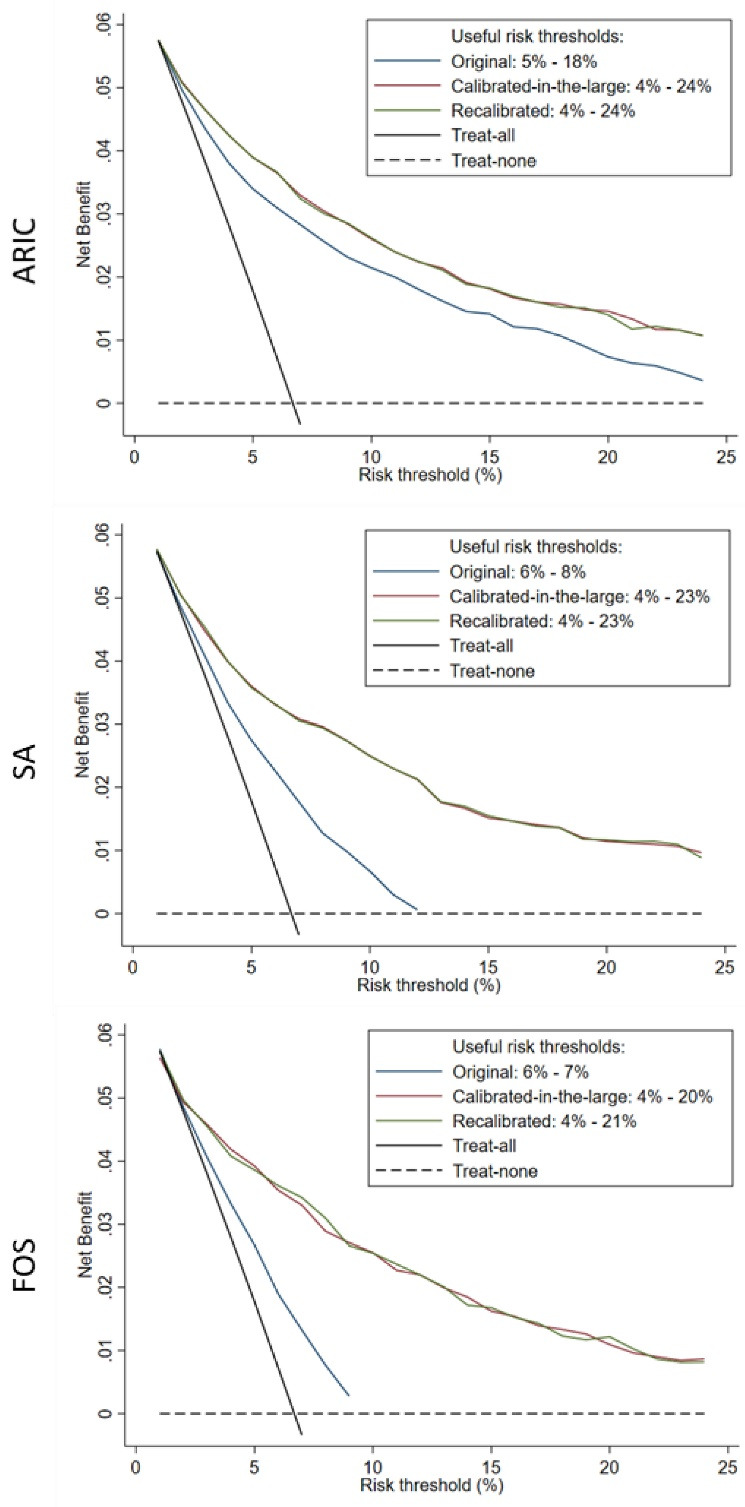
Decision Curves of Risk Models. The net benefit of the risk prediction models were compared with the net benefits of two different scenarios without screening: treat-none and treat-all. The risk prediction models were useful in a risk threshold if the differences between the net benefit of the risk prediction model and no-screening methods were equal or higher than 0.01; cut-off of 0.01 for the difference in the net benefit for minimal invasive screening methods has been suggested previously. Abbreviations: SA, Saint Antonio; FOS, Framingham Offspring Study; ARIC, Atherosclerosis Risk in Communities.

Figure 2 shows the decision curves of the risk prediction models. Calibrated-in-the-large models had considerably higher net benefits across the different risk thresholds as compared to the original models; however, the recalibrated models did not perform better than calibrated-in-the-large models. Based on the decision curves, the calibrated-in-the-large versions of SA, ARIC, and FOS prediction models were useful between risk thresholds of 4%-23%, 4%-24%, and 4%-21%, respectively. Comparable findings were observed in those with WtHR ≥0.55 and AUSDRISK >15 (Figure S4, Supplementary file 2). Based on the results from decision curves, predicted risk of ≥7.5%, ≥10%, ≥15%, and ≥20% were selected as cut-offs to be used in developing stepwise screening methods.

###  Stepwise Screening Methods

 Thirty-two stepwise screening methods were developed by combining a non-invasive measurement (WtHR ≥0.55 or AUSDRISK >15) with a lab-based measurement (FPG ≥5.0 mmol/L, ≥5.3 mmol/L, ≥5.5 mmol/L, and ≥6.1 mmol/L or predicted risk based on risk prediction models ≥7.5%, ≥10%, ≥15%, and ≥20%). Regarding the first step of stepwise methods based on WtHR, 49% of participants had WtHR ≥0.55 (95% CI: 47; 51), therefore, found eligible for participating for the second step of the screening test; the corresponding proportion for stepwise methods based on AUSDRISK >15 was 53% (95% CI: 51; 55). In other words, based on the first step of screening methods, 51% of participants in stepwise methods based on WtHR and 47% of participants in stepwise methods based on AUSDRISK had not high enough risk according to require the second step of the screening test that involves lab tests. The sensitivity, specificity, and PPV for the first step was 74.9% (95% CI: 68.3; 80.7), 50.8% (95% CI: 51.0-54.7), and 10.2% (95% CI: 8.7; 11.9) for stepwise methods based on WtHR and 78.3% (95% CI: 72.0; 83.8), 48.5% (95% CI: 46.6; 50.3), and 9.8% (95% CI: 8.4; 11.4) stepwise methods based on AUSDRISK, respectively.

Table 2 compares the sensitivity, specificity, and PPV of different stepwise methods of identifying individuals with 5-year incident type 2 diabetes. The sensitivity of the stepwise screening methods ranged from 20% in WtHR ≥0.55-FPG ≥6.1 mmol/L method to 68% in AUSDRISK >15-FPG ≥5.0 mmol/L method, and corresponding specificities ranged from 98% in AUSDRISK >15-FPG ≥5.0 mmol/L method to 70% in WtHR ≥0.55-FPG ≥6.1 mmol/L method. Based on the different stepwise screening methods, between 3% and 33% of the screened population would be eligible for structured lifestyle interventions. The risk of 5-year type 2 diabetes in those who screened positive (PPV) ranged from 14% to 35% based on the different screening methods.

 The performance of stepwise screening methods in the complete-case analyses and the analyses on data with imputed type 2 diabetes status were comparable with the original analyses (Tables S2 and S3, Supplementary file 2).

**Table 2:** Performance of Stepwise Methods in Identifying Individuals With 5-Year Incident Type 2 Diabetes

**Step 1**	**Step 2**	**Sensitivity% (95% CI)**	**Specificity% (95% CI)**	**PPV % (95% CI)**	**The Proportion of Screened Population Need Blood Test (95% CI)**	**The Proportion of Screened Population Need Intervention (95% CI)**
WtHR ≥0.55	FPG ≥5.0 mmol/L	66 (59; 72)	73 (71; 75)	15 (13; 17)	49 (47; 51)	30 (28; 31)
WtHR ≥0.55	FPG ≥5.3 mmol/L	58 (51; 65)	83 (82; 85)	20 (17; 23)	49 (47; 51)	20 (18; 21)
WtHR ≥0.55	FPG ≥5.5 mmol/L	47 (40; 54)	91 (90; 92)	27 (23; 32)	49 (47; 51)	11 (10; 13)
WtHR ≥0.55	FPG ≥6.1 mmol/L	20 (14; 26)	98 (98; 99)	46 (35; 56)	49 (47; 51)	3 (2; 4)
WtHR ≥0.55	SA ≥7.5%	60 (52.5; 66)	84 (82; 85)	21 (17; 24)	49 (47; 51)	19 (18; 21)
WtHR ≥0.55	SA ≥10%	53 (46; 60)	88 (87; 89)	24 (20; 28)	49 (47; 51)	15 (14; 16)
WtHR ≥0.55	SA ≥15%	41 (34; 48)	92 (91; 93)	27 (22; 32)	49 (47; 51)	10 (9; 11)
WtHR ≥0.55	SA ≥20%	33 (27; 40)	95 (94; 96)	33 (26; 39)	49 (47; 51)	7 (6; 8)
WtHR ≥0.55	ARIC ≥7.5%	59 (52; 66)	83 (82; 85)	20 (17; 24)	49 (47; 51)	19 (18; 21)
WtHR ≥0.55	ARIC ≥10%	53 (45; 60)	88 (87; 89)	24 (20; 28)	49 (47; 51)	15 (14; 16)
WtHR ≥0.55	ARIC ≥15%	42 (35; 49)	93 (92; 94)	29 (24; 35)	49 (47; 51)	10 (8; 11)
WtHR ≥0.55	ARIC ≥20%	35 (28; 42)	95 (95; 96)	35 (28; 41)	49 (47; 51)	7 (6; 8)
WtHR ≥0.55	FOS ≥7.5%	55 (48; 62)	86 (85; 87)	22 (18; 25)	49 (47; 51)	17 (16; 18)
WtHR ≥0.55	FOS ≥10%	51 (44; 58.5)	89 (88; 90)	25 (21; 30)	49 (47; 51)	14 (12; 15)
WtHR ≥0.55	FOS ≥15%	41 (34; 48)	92 (91; 93)	27 (22; 32)	49 (47; 51)	10 (9; 11)
WtHR ≥0.55	FOS ≥20%	36 (29; 43)	94 (93; 95)	31 (25; 37)	49 (47; 51)	8 (7; 9)
AUSDRISK >15	FPG ≥5.0 mmol/L	68 (62; 75)	70 (68; 71)	14 (12; 16)	53 (51; 55)	33 (31; 34)
AUSDRISK >15	FPG ≥5.3 mmol/L	61 (54; 68)	81 (79; 82)	18 (16; 22)	53 (51; 55)	22 (21; 23.5)
AUSDRISK >15	FPG ≥5.5 mmol/L	48 (41; 55)	89 (88; 91)	25 (20; 29)	53 (51; 55)	13 (12; 14)
AUSDRISK >15	FPG ≥6.1 mmol/L	22 (16; 28)	98 (97; 98)	43 (34; 53)	53 (51; 55)	3 (3; 4)
AUSDRISK >15	SA ≥7.5%	60 (53; 67)	82 (81; 83)	19 (16; 22)	53 (51; 55)	21 (19; 22)
AUSDRISK >15	SA ≥10%	53 (46; 60)	87 (85; 88)	22 (18; 26)	53 (51; 55)	16 (15; 17)
AUSDRISK >15	SA ≥15%	41 (35; 48)	91 (90; 92)	26 (21; 31)	53 (51; 55)	11 (10; 12)
AUSDRISK >15	SA ≥20%	34 (28; 41)	95 (94; 95)	32 (25; 38)	53 (51; 55)	7 (6; 8)
AUSDRISK >15	ARIC ≥7.5%	61 (54; 67.5)	82 (81; 84)	20 (16; 23)	53 (51; 55)	21 (19; 22)
AUSDRISK >15	ARIC ≥10%	52 (45; 59)	87 (86; 88)	22 (18; 26)	53 (51; 55)	16 (14; 17)
AUSDRISK >15	ARIC ≥15%	42 (35; 49)	92 (91; 93)	28 (23; 33)	53 (51; 55)	10 (9; 11)
AUSDRISK >15	ARIC ≥20%	35 (29; 42)	95 (94; 96)	34 (27; 40)	53 (51; 55)	7 (6; 8)
AUSDRISK >15	FOS ≥7.5%	57 (50; 63)	84 (83; 86)	21 (17; 24)	53 (51; 55)	18 (17; 20)
AUSDRISK >15	FOS ≥10%	53 (46; 60)	88 (87; 89)	24 (20; 29)	53 (51; 55)	15 (13; 16)
AUSDRISK >15	FOS ≥15%	42 (34.5; 49)	92 (91; 93)	26 (21; 31)	53 (51; 55)	11 (10; 12)
AUSDRISK >15	FOS ≥20%	36 (29; 43)	94 (93; 95)	29 (23.5; 35)	53 (51; 55)	30 (28; 31)

Abbreviations: WtHR, waist-to-height ratio; AUSDRISK, Australian Type 2 Diabetes Risk Assessment Tool; FPG; Fasting plasma glucose; SA, Saint Antonio; FOS, Framingham Offspring Study; ARIC, Atherosclerosis Risk in Communities; PPV, positive predictive value. Risk of developing type 2 diabetes within 5 years based on calibrated models equals to $\frac{1}{1+e^{-X}}$ where: In SA model, X = -14.714 + 0.028 * age (years) + 0.661 * (1 if female; 0 if male) + 0.481 * (1 if positive family history of type 2 diabetes; 0 if otherwise) + 0.079 * fasting plasma glucose (mg/dL) + 0.018 * systolic blood pressure (mm Hg) - 0.039 * HDL-C (mg/dL) + 0.070 * BMI (kg/m^2^). In ARIC model, X = -10.761 + 0.173 * age (years) + 0.4981 * (1 if positive family history of type 2 diabetes; 0 if otherwise) + 1.5849 * fasting plasma glucose (mmol/L) + 0.0111 * systolic blood pressure (mm Hg) + 0.0273 * waist circumference (cm) + 0.0326 * height (cm) - 0.4718 * HDL-C (mmol/L) + 0.2420 * Triglycerides (mmol/L). In FOS, X = -20.999 + log(0.99) * age (years) + log(0.65) * (1 if male; 0 if female) + log(1.55) * (1 if positive family history of type 2 diabetes; 0 if otherwise) + log(1.15) * fasting plasma glucose (mg/dL) + log(1.01) * systolic blood pressure (mm Hg) + log(0.96) * HDL-C (mg/dL) + log(1.05) * waist circumference (cm) + log(1.04) * BMI (kg/m^2^). AUSDRISK score = 3 * (1 if Male; 0 if otherwise) + 2 * (1 if aged between 35-44 years; 0 if otherwise) + 4 * (1 if aged between 45-54 years; 0 if otherwise) + 6 * (1 if aged between 55-64 years; 0 if otherwise) + 8 * (1 if aged ≥65 years; 0 if otherwise) + 2 * (1 if Middle Eastern; 0 if otherwise) + 3 * (1 if family history of diabetes (self-report); 0 if otherwise) + 6 * (1 if history of high blood glucose (self-report); 0 if otherwise) + 2 * (1 if use of blood pressure medication (self-report); 0 if otherwise) + 2 * (1 if current smoking (self-report); 0 if otherwise) + 2 * (1 if physically inactive (self-report); 0 if otherwise) + 3 * (1 if BMI between 25-29.9 (kg/m^2^); 0 if otherwise) + 6 * (1 if BMI between 30-34.9 (kg/m^2^); 0 if otherwise) + 8 * (1 if BMI ≥30 (kg/m^2^); 0 if otherwise) + 4 * (1 if WC between 90-99.9 cm in men or between 80-89.9 cm in women; 0 if otherwise) + 7 * (1 if WC ≥100 cm in men or ≥90 cm in women; 0 if otherwise).

## Discussion

###  Summary of Findings

 This is the first study that developed several stepwise methods for identifying those at high risk of type 2 diabetes and assessed their performance in Iranian population. This study also validated three well-known lab-based risk prediction models. We showed that, after calibration, lab-based prediction models are valid tools for the prediction of the risk of type 2 diabetes and using them in combination with non-invasive measurements can form effective stepwise screening methods with acceptable sensitivity (identifying up to 68% of individuals with 5-year incident type 2 diabetes) and specificity (excluding 70% of individuals without 5-year incident type 2 diabetes). Using stepwise methods can also eliminate the need for lab measurements in about half of the screened population and limit the proportion of the population who need preventive interventions to less than 35%.

###  Validation of Lab-Based Prediction Models

 In the current study, the discrimination power of the lab-based risk prediction models were good with AUCs >0.80; these findings are in line with previous studies that showed high discrimination power of these models (AUC >0.80) in different populations.^21,22,33-36^ High discrimination power of these models in the Iranian population indicates that lab measurements used in these models have high predictive power in this population.^28,36^ In regards with calibration, however, the original models overestimated the risk of 5-year incident type 2 diabetes in our study that was rectified after calibration of the intercept of the ARIC and SA models and calibration of intercept and slope of the FOS model.^28,36^ The lack of calibration in the original models might be rooted in differences in the follow-up period or ethnicity.^28,36^ All three models in these study were developed to predict the risk of the type 2 diabetes in studies with follow-up longer than 5 years (ranged 7-9 years)^19,20,23^ and using these models for predicting the risk of 5-year incident type 2 diabetes led to an overestimation of the risk.^28,36^ Moreover, previous studies showed the important role of ethnicity in the development of type 2 diabetes even accounting for other type 2 diabetes risk factors.^37^ Our findings also showed that by simple calibration of models, these prediction models can predict the risk of 5-year incident type 2 diabetes in the Iranian population with high validity. These findings were further supported by our net benefit assessment of the models that showed calibrated models are useful in a wide range of risk thresholds ranged from 4% to 21%.

###  Performance of Stepwise Screening Methods

 The stepwise methods developed in this study had acceptable performance in identifying those at high risk of developing type 2 diabetes with sensitivities ranging from 20% to 68% and specificities ranging from 70% to 98%; these findings are comparable with those of previous studies.^9,12,38^ In a study conducted by Lee et al,^9^ six different stepwise methods were developed by combining AUSDRISK prediction model and a blood test (FPG, hemoglobin A1c [HBA1c], or oral glucose tolerance test) with sensitivities ranging from 20% to 47% and specificities ranging from 81% to 98%.

 Given the fact that these screening strategies fail to identify between one-third to half of the participants with 5-year incident type 2 diabetes, one might suggest reducing the cut-offs for non-invasive or lab-based measurements to increase stepwise screening methods sensitivity. We, however, discourage reducing the cut-offs due to some problems involved in it. First, lower cut-offs increase the sensitivity of a test with the price of a reduction in its specificity and PPV, so it means that the population who screened positive based on the tests would have a lower risk of developing 5-year incident type 2 diabetes. There is strong evidence showing that the lifestyle intervention programs have limited effectiveness in individuals at low risk of developing type 2 diabetes and previous studies recommended to deliver these interventions to those with at least 20% risk of developing type 2 diabetes in short time.^1-5^ Therefore, we here recommend those stepwise screening methods with PPVs higher than 20%. Another problem with lowering cut-offs for stepwise screening methods is exposing further pressure on laboratory resources and the healthcare system as it requires further laboratory tests in the population and leads to a higher number of eligible participants, most of whom will be false positives. A practical and feasible solution to improve recruitment of individuals at high risk of type 2 diabetes is to use readily-available information collected during different medical procedures in addition to stepwise screening methods. For example, in the National Type 2 Diabetes Prevention Program in Finland,^7^ in addition to those who had a high risk of developing type 2 diabetes based on a stepwise screening method, people with a history of elevated blood glucose in their medical records or a history of gestational diabetes were recruited in the lifestyle interventions.

 We avoid recommending a particular stepwise screening method in our study. We instead recommend healthcare providers to select the best option based on the acceptability of screening methods, available intervention programs, and resources in their settings. For example, in case that the available preventive interventions are costly and resource-intensive, a screening method that selected lower proportion of the screened population with higher risk would be preferable, while in case of inexpensive interventions or settings with ample resources, screening methods with higher sensitivities would be preferable. One should also consider the potential impacts of the screening methods on the outcome of lifestyle interventions. Assuming that lifestyle intervention programs reduce the risk of type 2 diabetes by 30% in Iranian population - which is consistence with previous meta-analyses^1–5^-, using a stepwise screening method with a sensitivity of about 50% (such as WtHR ≥0.55-FPG ≥5.5 mmol/L) potentially can lead to a reduction in the incidence of type 2 diabetes in the whole population by 15%; while the corresponding impact would be 18% reduction in the incidence of type 2 diabetes in the whole population if a stepwise method with a sensitivity of about 60% (such as WtHR ≥0.55-SA ≥7.5%) will be used.^39^

 We here also highlight the fact that these screening methods for identifying those at high risk of type 2 diabetes do not eliminate the need for screening for other cardiometabolic risk factors or routine check-ups in the community. Undoubtedly, we need valid and reliable screening methods as well as effective interventions for other cardiometabolic risk factors to achieve ideal cardio-metabolic health.

###  Strengths and Limitations

 This study has some strengths and limitations. For strengths, to the best of our knowledge, it is the first study that developed stepwise screening methods for identifying those at high risk of type 2 diabetes in the LMIC and it is the first comparative validation study in Iran for these three well-known risk prediction models. The large sample size is one of the main strengths of this study, leading to high precision in our estimates. As for limitations, we had a drop-out rate of 27%. To address this limitation, we imputed the missing information in our sensitivity analyses; the findings of which were generally similar to those of original analyses. We used Hosmer–Lemeshow Chi-square to assess the calibration of the models, while some evidence showed that this test has a high rejection rate of acceptable models when large samples are used^40^; to address this limitation, we plotted predicted outcome probabilities (x-axis) against observed outcomes (y-axis) using a LOWESS line as recommended in guidelines by Transparent Reporting of a multivariable prediction model for Individual Prognosis or Diagnosis Initiative.^30^ Moreover, we did not use HbA1c cut points to define type 2 diabetes in this study. HbA1c did not measure in the different waves of TLGS because HbA1c was not introduced as a component for diagnosis of type 2 diabetes at the time of designing TLGS (year: 1999); it was costly measurement, and there was not a standardized method for measurement of HbA1c in Iranian population. This limitation might lead to missing a subgroup of people with type 2 diabetes with high HbA1C but normal FPG and 2hPG; however, this limitation hardly can affect the findings of this study since the previous studies showed that very small proportion of undiagnosed cases of type 2 diabetes have high HbA1c but normal FPG and 2hPG (~0.3%).^41^

## Conclusion

 In conclusion, lab-based prediction models are valid tools for the prediction of the risk of type 2 diabetes after calibration; and using them in combination with non-invasive measurements can form effective stepwise screening methods with acceptable sensitivity and specificity in Iranian population. Using stepwise methods can also eliminate the need for lab measurements in about half of the screened population and limit the proportion of the population who need preventive interventions.

## Ethical issues

 The Ethics Committee of the Research Institute for Endocrine Sciences, Shahid Beheshti University of Medical Sciences confirmed the design of the TLGS study and all participants provided written free and informed consent. The privacy rights of subjects have always been observed. The work described here has been carried out in accordance with The Code of Ethics of the World Medical Association (Declaration of Helsinki) for experiments involving humans.

## Competing interests

 Authors declare that they have no competing interests.

## Authors’ contributions

 Study conception and design: ML, DK. Acquisition of the data: FA, FH, and DK. Wwriting the first draft: ML. Analysis and interpretation of data: ML, MAM, and DK. Critical revision: ML, MAM, FA, FH, BO, and DK. Supervision: FA, MAM, BO, DK, and FH.

## Funding

 This work was supported by the National Research Council of the Islamic Republic of Iran [grant number 121] and also by Shahid Beheshti University of Medical Sciences.

## Role of the funding source

 The funding agreement ensured the authors’ independence in study design; in the collection, analysis and interpretation of data, in the writing of the report and in the decision to submit the article for publication.

## Supplementary files

Supplementary file 1. Brief Description of Risk Prediction Models.

Supplementary file 2 contains Figures S1-S4 and Tables S1- S3.
