# Supplementary material for: Performance of Stepwise Screening Methods in Identifying Individuals at High Risk of Type 2 Diabetes in an Iranian Population
Source: Int J Health Policy Manag. 2021 May 5;11(8):1391–400. doi: 10.34172/ijhpm.2021.22 (PMC9808334; doi:10.34172/ijhpm.2021.22)
Supplement: Supplementary file 1 — Brief Description of Risk Prediction Models. [file ijhpm-11-1391-s001.pdf]

Article title: Performance of Stepwise Screening Methods in Identifying Individuals at High Risk of Type 2 Diabetes in an Iranian Population

Journal name: International Journal of Health Policy and Management (IJHPM)

Authors' information: Mojtaba Lotfaliany1[,2,3,4], Farzad Hadaegh[5], Mohammad Ali Mansournia[6], Fereidoun Azizi[7], Brian Oldenburg[3,8], Davood Khalili[5,1]*

[1]Department of Biostatistics and Epidemiology, Research Institute for Endocrine Sciences, Shahid Beheshti University of Medical Sciences, Tehran, Iran.

[2]Barwon Health, Geelong, VIC, Australia.

[3]School of Population and Global Health, University of Melbourne, Melbourne, VIC, Australia.

[4]Institute for Mental and Physical Health and Clinical Translation (IMPACT), Deakin University, Geelong, VIC, Australia.

[5]Prevention of Metabolic Disorders Research Center, Research Institute for Endocrine Sciences, Shahid Beheshti University of Medical Sciences, Tehran, Iran.

[6]Department of Epidemiology and Biostatistics, School of Public Health, Tehran University of Medical Sciences, Tehran, Iran.

[7]Endocrine Research Center, Research Institute for Endocrine Sciences, Shahid Beheshti University of Medical Sciences, Tehran, Iran.

[8]WHO Collaborating Centre on Implementation Research for Prevention & Control of NCDs, University of Melbourne, Melbourne, VIC, Australia.

(*Corresponding author: dkhalili@endocrine.ac.ir)

Supplementary file 1: Brief Description of Risk Prediction Models

The Framingham Offspring Study (FOS) risk score for type 2 diabetes (1) was developed in a cohort of 3140 middle-aged (45-65 years old) who were free of type 2 diabetes based on the oral glucose tolerance test on the fifth clinic examination of the Framingham Offspring Study. The model has different versions (i.e. personal model, simple clinical model, and complex clinical models). The simple clinical model version with continuous variables was selected for this study as the more complex models needed laboratory tests such as HOMA-IR and Gutt insulin sensitivity index that are not routinely available in Iran. The simple clinical model uses age, gender, fasting plasma glucose, BMI, waist circumference, HDL cholesterol, systolic blood pressure, and parental history of diabetes to predict 7-year incident type 2 diabetes based on fasting blood glucose (1).

The Saint Antonio Diabetes Prediction Model (SA) was developed in a cohort of 1791 Mexican Americans and 1112 non-Hispanic whites without diabetes who aged between 25 and 64 years (2). The model uses age, sex, ethnicity, systolic blood pressure, HDL-C, BMI, family history of diabetes and FPG to predict the 7.5-year incident type 2 diabetes based on fasting plasma glucose level and 2-hour plasma glucose level (2).

Atherosclerosis Risk in Communities (ARIC) risk prediction model for type 2 diabetes was developed in ARIC cohort study in the US, using the data of 7915 adults aged between 45 and 65 (3). The ARIC risk prediction model has several versions: clinical information only, and clinical information + glucose, and clinical information + glucose+ lipids. We used the most elaborated version (i.e. clinical information + glucose+ lipids) due to its better discrimination power as compared with two other versions in the original article (3). This version of ARIC model uses age, race, parental history of diabetes, fasting glucose, systolic blood pressure, waist circumference, height, HDL cholesterol, and triglycerides to predict incidence of type 2 diabetes after 9 years of follow-up (3).

AUSDRISK developed in an Australian population aged ≥25 years without physician-diagnosed diabetes at baseline who were followed for 5 years (4). AUSDRISK uses age, sex, ethnicity, parental history of diabetes, history of high blood glucose level, use of antihypertensive medications, smoking, physical inactivity, and waist circumference to predict the incidence of type 2 diabetes based on fasting plasma glucose level and 2-hour plasma glucose level (4). In our previous study in TLGS population, we showed that AUSDRISK is valid tool for identifying those at high risk of type 2 diabetes with acceptable discrimination power (AUS: 0.77) and calibration(5).

References:

1. Wilson P, Meigs J, Sullivan L, … CF-A of internal, 2007 undefined. Prediction of incident diabetes mellitus in middle-aged adults: the Framingham Offspring Study. jamanetwork.com [Internet]. [cited 2018 Nov 27]; Available from: https://jamanetwork.com/journals/jamainternalmedicine/fullarticle/486842

2. Stern MP, Williams K, Haffner SM. Identification of persons at high risk for type 2 diabetes mellitus: do we need the oral glucose tolerance test? Ann Intern Med. 2002;136(8):575–81.

3. Schmidt M, Duncan B, Bang H, … JP-D, 2005 undefined. Identifying individuals at high risk for diabetes: The Atherosclerosis Risk in Communities study. Am Diabetes Assoc [Internet]. [cited 2018 Nov 27]; Available from: http://care.diabetesjournals.org/content/28/8/2013.short

4. Chen L, Magliano DJ, Balkau B, Colagiuri S, Zimmet PZ, Tonkin AM, et al. AUSDRISK: an Australian Type 2 Diabetes Risk Assessment Tool based on demographic, lifestyle and simple anthropometric measures. Med J Aust. 2010;192(4):197.

5. Lotfaliany M, Oldenburg B, Azizi F, Khalili D. Validation, Calibration, and Clinical Usefulness of Findrisc, Ausdrisk, and ADA Risk Scores for Detecting 5-Year Incident Type 2 Diabetes in Tehran Lipid and Glucose Study (TLGS). In: 17th SMDM. Leiden, The Netherlands: Society for Medical Decision Making; 2018. p. PS2-5.
