# Supplementary material for: Performance of Stepwise Screening Methods in Identifying Individuals at High Risk of Type 2 Diabetes in an Iranian Population
Source: Int J Health Policy Manag. 2021 May 5;11(8):1391–400. doi: 10.34172/ijhpm.2021.22 (PMC9808334; doi:10.34172/ijhpm.2021.22)
Supplement: Supplementary file 2 — contains Figures S1-S4 and Tables S1- S3. [file ijhpm-11-1391-s002.pdf]

Article title: Performance of Stepwise Screening Methods in Identifying Individuals at High Risk of Type 2 Diabetes in an Iranian Population

Journal name: International Journal of Health Policy and Management (IJHPM)

Authors' information: Mojtaba Lotfaliany1[,2,3,4], Farzad Hadaegh[5], Mohammad Ali Mansournia[6], Fereidoun Azizi[7], Brian Oldenburg[3,8], Davood Khalili[5,1]*

[1]Department of Biostatistics and Epidemiology, Research Institute for Endocrine Sciences, Shahid Beheshti University of Medical Sciences, Tehran, Iran.

[2]Barwon Health, Geelong, VIC, Australia.

[3]School of Population and Global Health, University of Melbourne, Melbourne, VIC, Australia.

[4]Institute for Mental and Physical Health and Clinical Translation (IMPACT), Deakin University, Geelong, VIC, Australia.

[5]Prevention of Metabolic Disorders Research Center, Research Institute for Endocrine Sciences, Shahid Beheshti University of Medical Sciences, Tehran, Iran.

[6]Department of Epidemiology and Biostatistics, School of Public Health, Tehran University of Medical Sciences, Tehran, Iran.

[7]Endocrine Research Center, Research Institute for Endocrine Sciences, Shahid Beheshti University of Medical Sciences, Tehran, Iran.

[8]WHO Collaborating Centre on Implementation Research for Prevention & Control of NCDs, University of Melbourne, Melbourne, VIC, Australia.

(*Corresponding author: dkhalili@endocrine.ac.ir)

Supplementary file 2

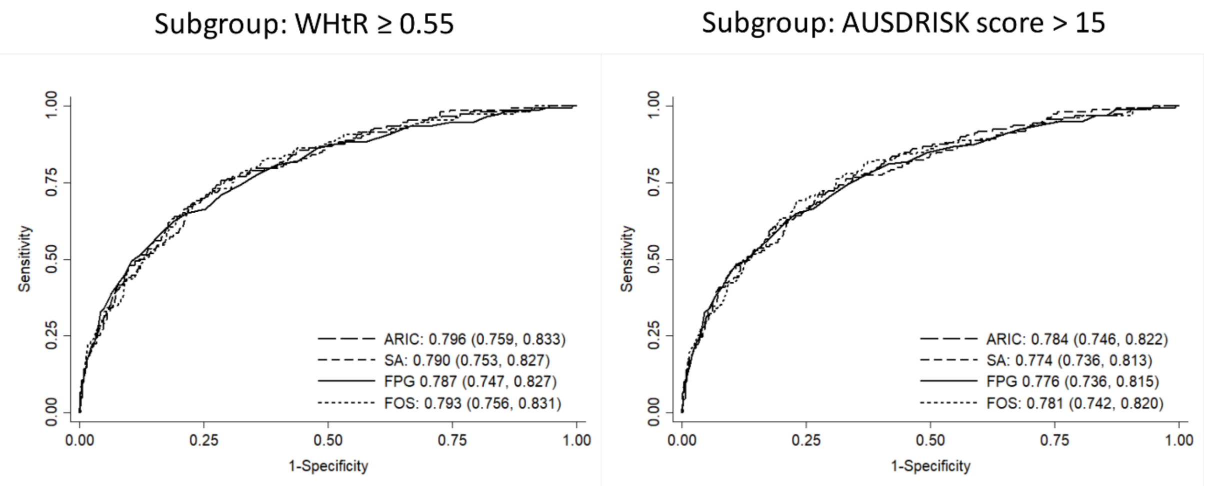

Figure S1

Title: ROC curves in those with WtHR ≥0.55 and AUSDRISK >15

Legend: ROC, Receiver operating characteristic; SA, Saint Antonio; FOS, Framingham Offspring Study; FPG, Fasting plasma glucose; ARIC, Atherosclerosis Risk in Communities.

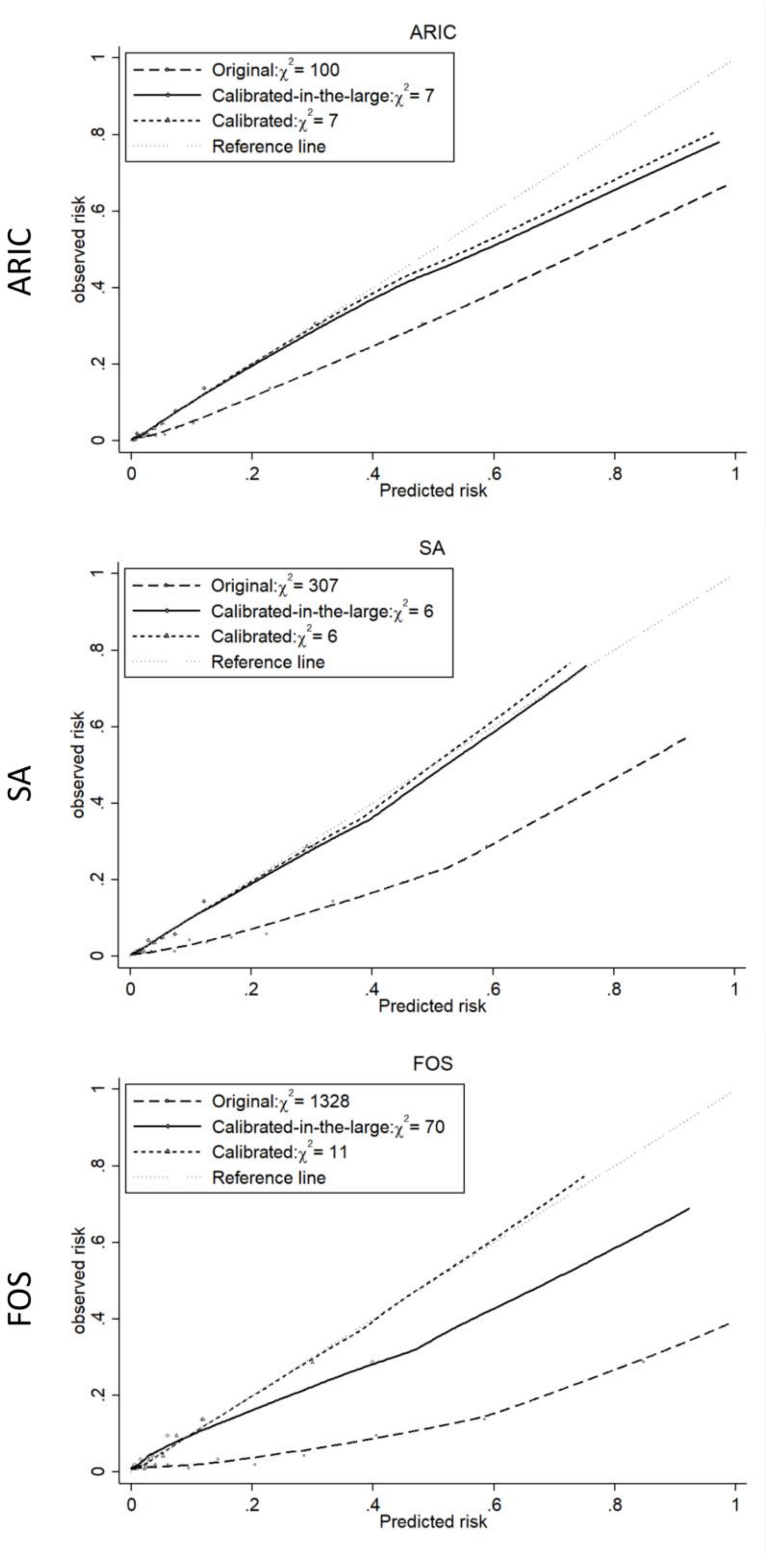

Figure S2

Title: Calibration plots.

Legend: SA, Saint Antonio; FOS, Framingham Offspring Study; ARIC, Atherosclerosis Risk in Communities.

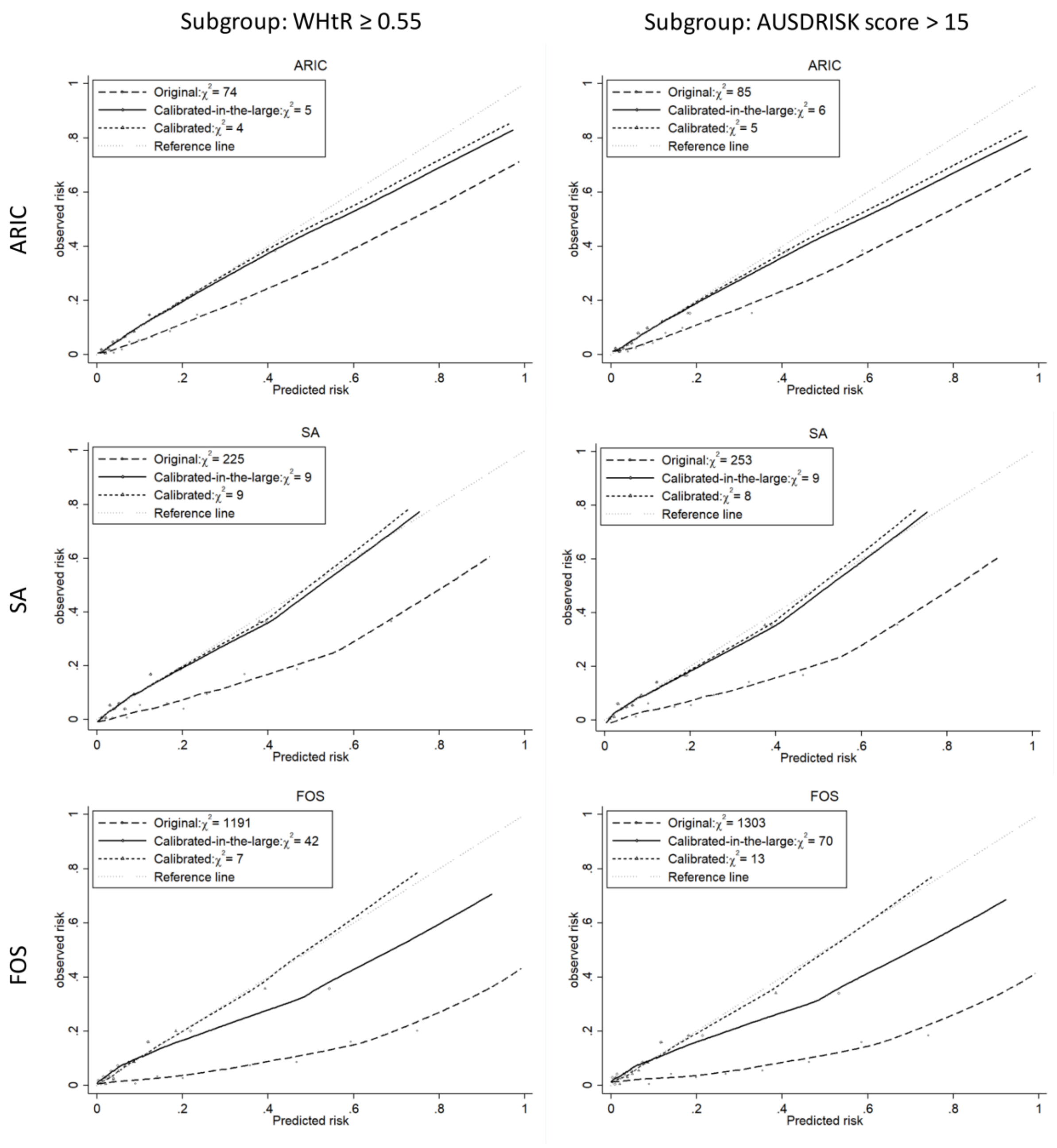

Figure S3

Title: Calibration plots in those with WtHR ≥0.55 and AUSDRISK >15

Legend: SA, Saint Antonio; FOS, Framingham Offspring Study; ARIC, Atherosclerosis Risk in Communities.

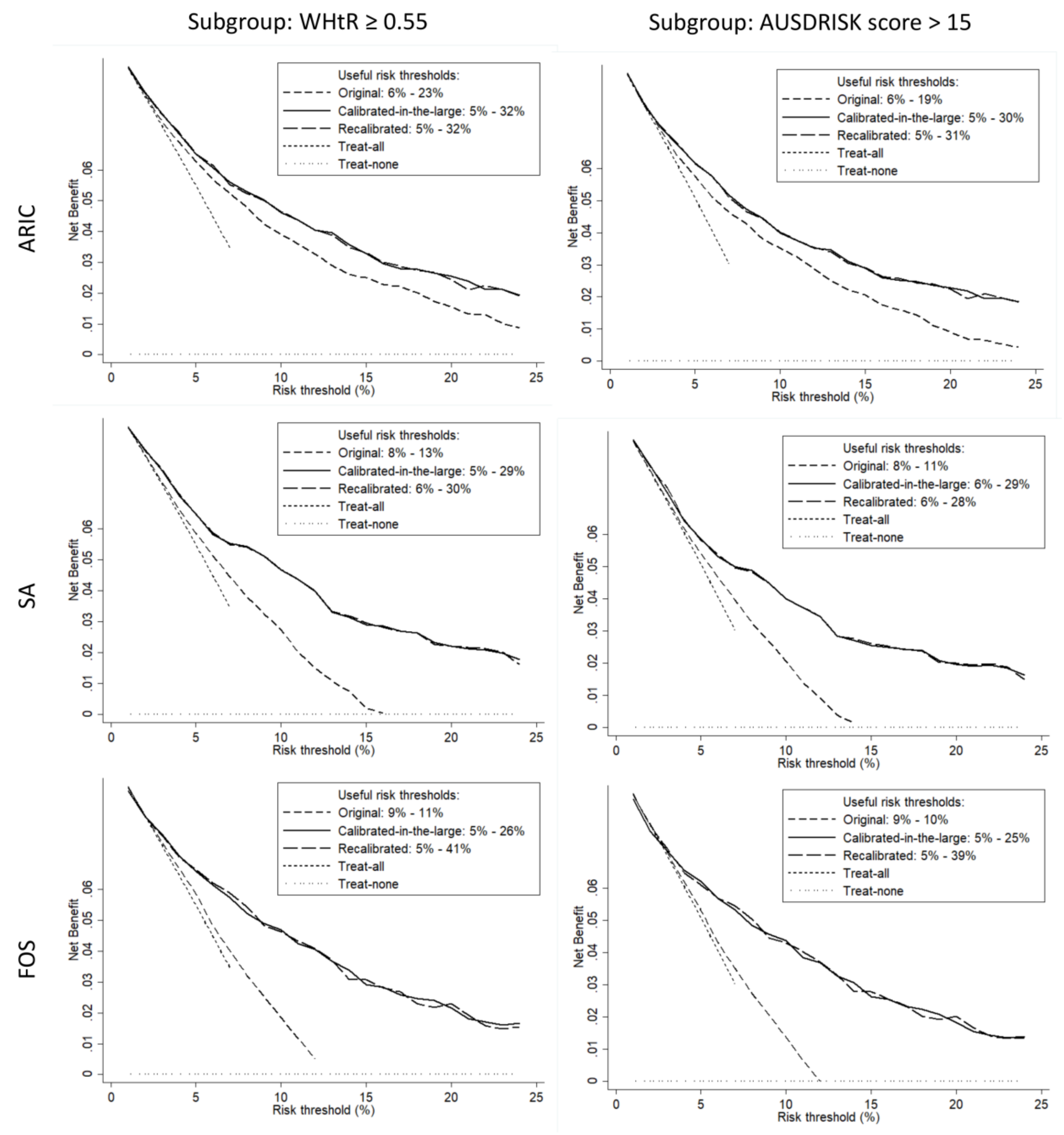

Figure S4:

Title: Decision Curves of Risk Models in those with WtHR ≥0.55 and AUSDRISK >15

Legend: SA, Saint Antonio; FOS, Framingham Offspring Study; ARIC, Atherosclerosis Risk in Communities.

Supplementary Table S1. Equation of the original and calibrated models.

Risk of developing type 2 diabetes within 5 years equals to $\frac{1}{1+e^{-X}}$ where X is:

**Risk prediction model**	**Equation**
**ARIC**	
Original model:	$X = -9.981 + \sum \beta_{x_i}\text{ ARIC}$
Calibrated-in-the-large models:	$X = -10.761 + \sum \beta_{x_i}\text{ ARIC}$
Recalibrated:	$X = -10.342 + 0.952 * \sum \beta_{x_i}\text{ ARIC}$
**SA**	
Original model:	$X = -13.415 + \sum \beta_{x_i}\text{ SA}$
Calibrated-in-the-large models:	$X = -14.714 + \sum \beta_{x_i}\text{ SA}$
Recalibrated:	$X = -14.174 + 0.957 * \sum \beta_{x_i}\text{ SA}$
**FOS**	
Original model:	$X = -18.607 + \sum \beta_{x_i}\text{ FOS}$
Calibrated-in-the-large models:	$X = -20.999 + \sum \beta_{x_i}\text{ FOS}$
Recalibrated:	$X = -15.067 + 0.689 * \sum \beta_{x_i}\text{ FOS}$

Where:

$$\sum \beta_{x_i}\text{ ARIC} = 0.173 * \text{age (years)} + 0.4981 * (\text{1 if positive family history of type 2 diabetes ; 0 if otherwise}) + 1.5849 * \text{fasting plasma glucose}\left(\frac{\text{mmol}}{\text{L}}\right) + 0.0111 * \text{systolic blood pressure (mmHg)} + 0.0273 * \text{waist circumference (cm)} + 0.0326 * \text{height (cm)} - 0.4718 * \text{HDL} - \text{C}\left(\frac{\text{mmol}}{\text{L}}\right) + 0.2420 * \text{Triglycerides}\left(\frac{\text{mmol}}{\text{L}}\right)$$

$$\sum \beta_{x_i}\text{ SA} = 0.028 * \text{age (years)} + 0.661 * (\text{1 if female ; 0 if male}) + 0.481 * (\text{1 if positive family history of type 2 diabetes ; 0 if otherwise}) + 0.079 * \text{fasting plasma glucose}\left(\frac{\text{mg}}{\text{dl}}\right) + 0.018 * \text{systolic blood pressure (mmHg)} - 0.039 * \text{HDL} - \text{C}\left(\frac{\text{mg}}{\text{dl}}\right) + 0.070 * \text{BMI}\left(\frac{\text{Kg}}{\text{m}^2}\right)$$

$$\sum \beta_{x_i}\text{ FOS} = \log(0.99) * \text{age (years)} + \log(0.65) * (\text{1 if male ; 0 if female}) + \log(1.55) * (\text{1 if positive family history of type 2 diabetes ; 0 if otherwise}) + \log(1.15) * \text{fasting plasma glucose}\left(\frac{\text{mg}}{\text{dl}}\right) + \log(1.01) * \text{systolic blood pressure (mmHg)} + \log(0.96) * \text{HDL} - \text{C}\left(\frac{\text{mg}}{\text{dl}}\right) + \log(1.00) * \text{Triglycerides}\left(\frac{\text{mg}}{\text{dl}}\right) + \log(1.05) * \text{waist circumference (cm)} + \log(1.04) * \text{BMI}\left(\frac{\text{Kg}}{\text{m}^2}\right)$$

Supplementary Table S2. Performance of stepwise methods in identifying individuals with 5-year incident type 2 diabetes in complete-case dataset.

Step 1	Step 2	Sensitivity (%)	Specificity (%)	PPV (%)	Proportion of screened population need blood test (%)	Proportion of screened population need intervention (%)
WHtR ≥ 0.55	FPG ≥ 5.0 mmol/L	67 (60; 73.5)	73 (71; 74)	14 (12; 17)	49 (47; 51)	30 (28; 31)
WHtR ≥ 0.55	FPG ≥ 5.3 mmol/L	59 (52; 66)	83 (82; 84)	19 (16; 23)	49 (47; 51)	20 (18; 21)
WHtR ≥ 0.55	FPG ≥ 5.5 mmol/L	48 (41; 56)	91 (90; 92)	27 (22; 32)	49 (47; 51)	12 (10; 13)
WHtR ≥ 0.55	FPG ≥ 6.1 mmol/L	22 (16; 28)	98 (98; 99)	48 (39; 59)	49 (47; 51)	3 (2; 4)
WHtR ≥ 0.55	SA ≥ 7.5%	60 (53; 67)	84 (83; 86)	21 (17; 24)	49 (47; 51)	19 (17; 20)
WHtR ≥ 0.55	SA ≥ 10%	54 (47; 61)	88 (87; 89)	24 (20; 28)	49 (47; 51)	15 (13; 16)
WHtR ≥ 0.55	SA ≥ 15%	41 (33; 48)	92 (91; 93)	27 (22; 33)	49 (47; 51)	10 (9; 11)
WHtR ≥ 0.55	SA ≥ 20%	32 (26; 39)	95 (95; 96)	33 (26; 40)	49 (47; 51)	6 (6; 7)
WHtR ≥ 0.55	ARIC ≥ 7.5%	59 (52; 66)	84 (82; 85)	20 (17; 24)	49 (47; 51)	19 (18; 20)
WHtR ≥ 0.55	ARIC ≥ 10%	53 (46; 60)	88 (87; 89)	24 (19; 28)	49 (47; 51)	14 (13; 16)
WHtR ≥ 0.55	ARIC ≥ 15%	42 (35; 49)	93 (92; 94)	29 (24; 34.5)	49 (47; 51)	9 (8; 10)
WHtR ≥ 0.55	ARIC ≥ 20%	34 (27.5; 41)	96 (95; 96)	35 (29; 43)	49 (47; 51)	6 (5; 7)
WHtR ≥ 0.55	FOS ≥ 7.5%	55 (48; 62)	86 (85; 87)	21 (18; 25)	49 (47; 51)	17 (15; 18)
WHtR ≥ 0.55	FOS ≥ 10%	50 (43; 57.5)	89 (88; 90)	24 (20; 29)	49 (47; 51)	13 (12; 15)
WHtR ≥ 0.55	FOS ≥ 15%	42 (34.5; 49)	92 (91; 93)	27 (22; 32.5)	49 (47; 51)	10 (9; 11)
WHtR ≥ 0.55	FOS ≥ 20%	35 (28; 42)	95 (94; 95)	31 (25; 38)	49 (47; 51)	7 (7; 8)
AUSDRISC > 15	FPG ≥ 5.0 mmol/L	70 (63; 76)	69 (68; 71)	14 (12; 16)	54 (52; 56)	33 (31; 35)
AUSDRISC > 15	FPG ≥ 5.3 mmol/L	63 (56; 70)	80 (79; 82)	18 (15; 21)	54 (52; 56)	22 (21; 24)
AUSDRISC > 15	FPG ≥ 5.5 mmol/L	50 (43; 58)	89 (88; 90)	24 (20; 29)	54 (52; 56)	13 (12; 15)
AUSDRISC > 15	FPG ≥ 6.1 mmol/L	24 (18.5; 31)	98 (97; 99)	45 (36; 56)	54 (52; 56)	3 (3; 4)
AUSDRISC > 15	SA ≥ 7.5%	62 (54; 69)	82 (81; 84)	19 (16; 23)	54 (52; 56)	20 (19; 22)
AUSDRISC > 15	SA ≥ 10%	55 (47; 61)	87 (86; 88)	22 (18.5; 26)	54 (52; 56)	16 (14; 17)
AUSDRISC > 15	SA ≥ 15%	42 (35; 49)	92 (91; 93)	26 (22; 31)	54 (52; 56)	10 (9; 11)
AUSDRISC > 15	SA ≥ 20%	34 (27; 41)	95 (94; 96)	32 (26; 39)	54 (52; 56)	7 (6; 8)
AUSDRISC > 15	ARIC ≥ 7.5%	61 (53; 67)	82 (81; 84)	19 (16; 22)	54 (52; 56)	20 (19; 22)
AUSDRISC > 15	ARIC ≥ 10%	53 (45; 60)	87 (86; 88)	22 (18; 26)	54 (52; 56)	15 (14; 17)
AUSDRISC > 15	ARIC ≥ 15%	42 (35.5; 50)	93 (91.5; 93)	28 (23; 33)	54 (52; 56)	10 (9; 11)
AUSDRISC > 15	ARIC ≥ 20%	36 (29; 43)	95 (95; 96)	35 (29; 42)	54 (52; 56)	7 (6; 7.5)
AUSDRISC > 15	FOS ≥ 7.5%	58 (51; 65)	85 (83; 86)	21 (17; 24)	54 (52; 56)	18 (17; 19.5)
AUSDRISC > 15	FOS ≥ 10%	53 (46; 60)	88 (87; 89)	24 (20; 28)	54 (52; 56)	14 (13; 16)
AUSDRISC > 15	FOS ≥ 15%	43 (36; 50.5)	92 (91; 93)	26 (21; 32)	54 (52; 56)	11 (10; 12)
AUSDRISC > 15	FOS ≥ 20%	36 (29; 43)	94 (93; 95)	30 (24; 37)	54 (52; 56)	8 (7; 9)

Risk of developing type 2 diabetes within 5 years based on calibrated models equals to $\frac{1}{1+e^{-X}}$ where:

In SA model, X= -14.714+ 0.028 *age (years)+0.661 * (1 if female ;0 if male)+0.481 * (1 if positive family history of type 2 diabetes ;0 if otherwise)+ 0.079 *fasting plasma glucose (mg/dl)+0.018* systolic blood pressure (mmHg)- 0.039 *HDL-C (mg/dl)+ 0.070*BMI (Kg/m^2)

In ARIC model, X = -10.761+ 0.173 *age (years) + 0.4981 * (1 if positive family history of type 2 diabetes; 0 if otherwise)+ 1.5849 * fasting plasma glucose (mmol/L) + 0.0111* systolic blood pressure (mmHg)+ 0.0273 * waist circumference (cm) + 0.0326 *height (cm) - 0.4718 *HDL-C (mmol/L) + 0.2420*Triglycerides (mmol/L)

In FOS, X= -20.999 + log(0.99) *age (years)+log(0.65)* (1 if male ;0 if female)+log(1.55)* (1 if positive family history of type 2 diabetes ;0 if otherwise)+ log(1.15)*fasting plasma glucose (mg/dl)+log(1.01)* systolic blood pressure (mmHg)+log(0.96)*HDL-C (mg/dl)+ log(1.05)* waist circumference (cm)+log(1.04)*BMI (Kg/m^2)

AUSDRISC score = 3 *(1 if Male;0 if otherwise)+ 2 *(1 if aged between 35-44 years;0 if otherwise) + 4 *(1 if aged between 45-54 years;0 if otherwise) +6*(1 if aged between 55-64 years;0 if otherwise)+ 8* (1 if aged ≥65 years;0 if otherwise)+ 2*(1 if Middle Eastern;0 if otherwise) + 3*(1 if family history of diabetes (self-report);0 if otherwise)+6 *(1 if history of high blood glucose (self-

report);0 if otherwise)+2 *(1 if use of blood pressure medication(self-report);0 if otherwise)+2 * (1 if current smoking (self-report);0 if otherwise)+2 * (1 if physically inactive (self-report);0 if otherwise)+ 3 * (1 if BMI between 25-29.9 (kg/m^2);0 if otherwise)+ 6 * (1 if BMI between 30-34.9 (kg/m^2);0 if otherwise)+ 8 *(1 if BMI ≥30 (kg/m^2);0 if otherwise)+4 * (1 if WC between 90-99.9 cm in men or between 80-89.9 cm in women ;0 if otherwise)+ 7 *(1 if WC ≥100 cm in men or ≥90 cm in women ;0 if otherwise)

Supplementary Table S3. Performance of stepwise methods in identifying individuals with 5-year incident type 2 diabetes in dataset with imputed variables and type 2 diabetes status.

Step 1	Step 2	Sensitivity (%)	Specificity (%)	PPV (%)	Proportion of screened population need blood test (%)	Proportion of screened population need intervention (%)
WHtR ≥ 0.55	FPG ≥ 5.0 mmol/L	59 (56; 63)	77 (75; 78)	35 (33; 38)	49 (48; 51)	30 (28; 31)
WHtR ≥ 0.55	FPG ≥ 5.3 mmol/L	50 (46; 53)	86 (85; 88)	44 (41; 48)	49 (48; 51)	20 (19; 21)
WHtR ≥ 0.55	FPG ≥ 5.5 mmol/L	36 (32; 39)	94 (93; 94)	55 (50.5; 59)	49 (48; 51)	12 (11; 13)
WHtR ≥ 0.55	FPG ≥ 6.1 mmol/L	14 (12; 17)	99 (99; 99)	79 (72; 85.5)	49 (48; 51)	3 (3; 4)
WHtR ≥ 0.55	SA ≥ 7.5%	49 (45; 52)	86 (85; 87)	43 (39; 46)	49 (48; 51)	20 (19; 22)
WHtR ≥ 0.55	SA ≥ 10%	44 (40; 48)	90 (89; 91)	49 (45; 53)	49 (48; 51)	16 (15; 17)
WHtR ≥ 0.55	SA ≥ 15%	34 (30; 37)	95 (94; 95)	58 (54; 63)	49 (48; 51)	10 (9; 11)
WHtR ≥ 0.55	SA ≥ 20%	25 (22; 28)	97 (96; 97)	63 (57; 68)	49 (48; 51)	7 (6; 8)
WHtR ≥ 0.55	ARIC ≥ 7.5%	50 (46; 53)	86 (85; 87)	44 (41; 47)	49 (48; 51)	20 (19; 21)
WHtR ≥ 0.55	ARIC ≥ 10%	44 (40; 47)	91 (90; 92)	51 (47; 55)	49 (48; 51)	15 (14; 16)
WHtR ≥ 0.55	ARIC ≥ 15%	32 (29; 35)	95 (94; 96)	58 (53; 63)	49 (48; 51)	10 (9; 11)
WHtR ≥ 0.55	ARIC ≥ 20%	25 (22; 28)	97 (96; 97)	63 (58; 69)	49 (48; 51)	7 (6; 8)
WHtR ≥ 0.55	FOS ≥ 7.5%	47 (43; 50)	89 (88; 90)	48 (45; 52)	49 (48; 51)	17 (16; 18)
WHtR ≥ 0.55	FOS ≥ 10%	40 (36; 43)	92 (91; 93)	52 (48.5; 56)	49 (48; 51)	14 (12; 15)
WHtR ≥ 0.55	FOS ≥ 15%	32 (29; 36)	95 (94; 96)	57 (52.5; 62)	49 (48; 51)	10 (9; 11)
WHtR ≥ 0.55	FOS ≥ 20%	28 (25; 31)	97 (96; 97)	64 (58.5; 69)	49 (48; 51)	8 (7; 9)
AUSDRISC > 15	FPG ≥ 5.0 mmol/L	64 (61; 68)	74 (72; 75)	35 (32; 37)	55 (53; 56)	33 (32; 35)
AUSDRISC > 15	FPG ≥ 5.3 mmol/L	54 (50; 58)	84 (83; 85)	43 (40; 46)	55 (53; 56)	23 (21; 24)
AUSDRISC > 15	FPG ≥ 5.5 mmol/L	40 (36; 43)	92 (91; 93)	53 (49; 57)	55 (53; 56)	13 (12; 14)
AUSDRISC > 15	FPG ≥ 6.1 mmol/L	17 (14; 19)	99 (99; 99)	77 (70; 84)	55 (53; 56)	4 (3; 4)
AUSDRISC > 15	SA ≥ 7.5%	52 (49; 56)	84 (83; 85)	42 (39; 45)	55 (53; 56)	22 (21; 24)
AUSDRISC > 15	SA ≥ 10%	46 (42; 50)	89 (88; 90)	47 (43.5; 51)	55 (53; 56)	17 (16; 19)
AUSDRISC > 15	SA ≥ 15%	35 (32; 39)	94 (93; 95)	57 (52; 62)	55 (53; 56)	11 (10; 12)
AUSDRISC > 15	SA ≥ 20%	27 (24; 30)	96 (96; 97)	62 (56; 67)	55 (53; 56)	8 (7; 9)
AUSDRISC > 15	ARIC ≥ 7.5%	53 (50; 57)	85 (84; 86)	44 (40; 47)	55 (53; 56)	22 (21; 23)
AUSDRISC > 15	ARIC ≥ 10%	46 (43; 49)	90 (89; 91)	50 (46; 54)	55 (53; 56)	17 (15; 18)
AUSDRISC > 15	ARIC ≥ 15%	34 (31; 38)	95 (94; 95)	58 (53; 63)	55 (53; 56)	11 (10; 11)
AUSDRISC > 15	ARIC ≥ 20%	27 (24; 30)	97 (96; 97)	64 (58.5; 69)	55 (53; 56)	7 (7; 8)
AUSDRISC > 15	FOS ≥ 7.5%	51 (47; 54)	88 (87; 89)	48 (45; 52)	55 (53; 56)	19 (18; 20)
AUSDRISC > 15	FOS ≥ 10%	43 (39; 46)	91 (90; 92)	52 (48; 56)	55 (53; 56)	15 (14; 16)
AUSDRISC > 15	FOS ≥ 15%	34 (31; 38)	94 (94; 95)	57 (53; 62)	55 (53; 56)	11 (10; 12)
AUSDRISC > 15	FOS ≥ 20%	30 (27; 33)	96 (96; 97)	64 (59; 69)	55 (53; 56)	8 (7.5; 9)

Risk of developing type 2 diabetes within 5 years based on calibrated models equals to $\frac{1}{1+e^{-X}}$ where:

In SA model, X= -14.714+ 0.028 *age (years)+0.661 * (1 if female ;0 if male)+0.481 * (1 if positive family history of type 2 diabetes ;0 if otherwise)+ 0.079 *fasting plasma glucose (mg/dl)+0.018* systolic blood pressure (mmHg)- 0.039 *HDL-C (mg/dl)+ 0.070*BMI (Kg/m^2)

In ARIC model, X = -10.761+ 0.173 *age (years) + 0.4981 * (1 if positive family history of type 2 diabetes; 0 if otherwise)+ 1.5849 * fasting plasma glucose (mmol/L) + 0.0111* systolic blood pressure (mmHg)+ 0.0273 * waist circumference (cm) + 0.0326 *height (cm) - 0.4718 *HDL-C (mmol/L) + 0.2420*Triglycerides (mmol/L)

In FOS, X= -20.999 + log(0.99) *age (years)+log(0.65)* (1 if male ;0 if female)+log(1.55)* (1 if positive family history of type 2 diabetes ;0 if otherwise)+ log(1.15)*fasting plasma glucose (mg/dl)+log(1.01)* systolic blood pressure (mmHg)+log(0.96)*HDL-C (mg/dl)+ log(1.05)* waist circumference (cm)+log(1.04)*BMI (Kg/m^2)

AUSDRISC score = 3 *(1 if Male;0 if otherwise)+ 2 *(1 if aged between 35-44 years;0 if otherwise) + 4 *(1 if aged between 45-54 years;0 if otherwise) +6*(1 if aged between 55-64 years;0 if otherwise)+ 8* (1 if aged ≥65 years;0 if otherwise)+ 2*(1 if Middle Eastern;0 if otherwise) + 3*(1 if

family history of diabetes (self-report);0 if otherwise)+6 *(1 if history of high blood glucose (self-report);0 if otherwise)+2 *(1 if use of blood pressure medication(self-report);0 if otherwise)+2 * (1 if current smoking (self-report);0 if otherwise)+2 * (1 if physically inactive (self-report);0 if otherwise)+ 3 * (1 if BMI between 25-29.9 (kg/m^2);0 if otherwise)+ 6 * (1 if BMI between 30-34.9 (kg/m^2);0 if otherwise)+ 8 *(1 if BMI ≥30 (kg/m^2);0 if otherwise)+4 * (1 if WC between 90-99.9 cm in men or between 80-89.9 cm in women ;0 if otherwise)+ 7 *(1 if WC ≥100 cm in men or ≥90 cm in women ;0 if otherwise)
